# Accelerating antiviral drug discovery: early hazard detection with a dual zebrafish and cell culture screen of a 403 compound library

**DOI:** 10.1007/s00204-024-03948-3

**Published:** 2024-12-27

**Authors:** Lisa Truong, Andrew A. Bieberich, Raymond O. Fatig, Bartek Rajwa, Michael T. Simonich, Robyn L. Tanguay

**Affiliations:** 1 Department of Environmental and Molecular Toxicology, Sinnhuber Aquatic Research Laboratory, Oregon State University, Corvallis, OR 97333 USA; 2 AsedaSciences Inc., West Lafayette, IN USA; 3 Bindley Bioscience Center, Purdue University, West Lafayette, IN 47907 USA

**Keywords:** Antiviral drug discovery, High throughput, SYSTEMETRIC® Cell Health Screen, Embryonic zebrafish model (ZBEscreen), Whole organism, Developmental neurotoxicity (DNT)

## Abstract

The constant emergence of new viral pathogens underscores the need for continually evolving, effective antiviral drugs. A key challenge is identifying compounds that are both efficacious and safe, as many candidates fail during development due to unforeseen toxicity. To address this, the embryonic zebrafish morphology, mortality, and behavior (ZBE) screen and the SYSTEMETRIC® Cell Health Screen (CHS) were employed to evaluate the safety of 403 compounds from the Cayman Antiviral Screening Library. Of these compounds, 114 were FDA-approved, 17 were discontinued, and 97 remained on the market. CHS identified 25% (104 compounds) as toxic, with a Cell Health Index™ (CHI) > 0.5. The embryonic zebrafish model identified an additional 20% as toxic (79), bringing the total to 183. ZBEscreen flagged 19 toxic hits among compounds still on the market, seven of which were also identified by CHS. The combined use of CHS and zebrafish models enhanced hazard detection. Together, CHS and ZBEscreen identified 45.5% of the library as potentially hazardous. Notably, the zebrafish non-hazardous compounds correlated strongly with over-the-counter or prescribed antiviral drugs, confirming their known safety profile. Over 130 hazard-associated compounds warranted further investigation. Using self-organizing maps, six distinct neighborhoods of compound similarity were identified. This dual approach streamlined the early detection of hazards associated with promising leads and is expected to facilitate faster, safer antiviral discovery.

**Supplementary Information:**

The online version contains supplementary material available at 10.1007/s00204-024-03948-3.

## Introduction

The emergence of new viral pathogens, exemplified by the global COVID-19 pandemic, illustrates the critical necessity for effective and safe antiviral drugs. The ability of viruses to rapidly mutate and cross species barriers adds layers of complexity to the challenge of developing timely and effective antiviral therapies. This urgency is compounded by the ongoing threat of established viruses such as HIV, influenza, and hepatitis B and C viruses, which continue to demand innovative solutions to prevent widespread morbidity and mortality (Balasubramaniam et al. 2021). A robust pipeline is needed to identify new antivirals that can swiftly bring safe and effective drugs from the laboratory to the clinic.

The development of antiviral drugs has significant hurdles, beginning with identifying compounds that demonstrate both efficacy and safety. Initial in vitro testing is the cornerstone for assessing a compound’s potential therapeutic benefits and risks (Niles et al. 2009). Still, many candidates fail due to unforeseen toxicity. The transition from successful in vitro results to safe and effective human trials is a critical leap and the crux of the high attrition rate in drug development (Hingorani et al. 2019; Sun et al. 2022). Such failures are hugely expensive and delay the advancement of antiviral therapies. The challenge lies in identifying potent antiviral compounds and detecting any safety liabilities as early in the discovery pipeline as possible. Scrapping a lead with a poor safety profile before it fails in mammalian or clinical testing would avoid millions of dollars in unnecessary costs and quickly refocus the pipeline on better leads.

The AsedaSciences SYSTEMETRIC® Cell Health Screen (CHS) provides a streamlined approach to cell-based phenotypic screening in drug discovery. This system automates the detection of predictive features in multiparametric flow-cytometry data using advanced data-processing methods. Feature extraction and a multidimensional classifier address some limitations of traditional cytometry analysis, such as the dependence on conventional gating techniques, enabling early assessment of a compound’s potential toxicity. The Cell Health Index™ (CHI) is used to identify high-risk compounds, supporting the selection of antiviral candidates for further evaluation. The CHS classifer is trained on a dataset comprising on-market, withdrawn and discontinued drugs, and compounds with known human toxicity, enabling a data-driven assessment of potential antiviral agents with the aim of improving the predictability of clinical outcomes.

The embryonic zebrafish model complements the CHS by providing a valuable whole organism approach to antiviral drug discovery. The zebrafish offers several advantages, including rapid development, transparency during embryonic stages, and genetic and physiologic similarities to humans, making it a useful model for evaluating both drug efficacy and toxicity. In addition, its suitability for high-throughput screening (HTS) technologies enables efficient, cost-effective testing of large compound libraries in a short time frame. The zebrafish model allows researchers to assess the effects of antiviral compounds on organ development and function, together with behavioral phenotypes indicative of developmental neurotoxicity, within days, facilitating the identification of potential candidates.

By harnessing the synergistic potential of the AsedaSciences SYSTEMETRIC® CHS and the embryonic zebrafish model, this study represents a paradigm shift in antiviral drug development. The strengths of these platforms allowed for a thorough and efficient evaluation of a library of 403 antiviral compounds, facilitating the early detection of potential toxic effects. This integrated approach may help to accelerate the identification of promising antiviral therapies while mitigating risks and costs associated with the drug development process.

## Methods

### Cayman antiviral library

The study utilized an antiviral compound library comprising 403 distinct chemicals obtained from the Cayman Chemical Company’s Antiviral Screening Library (Catalog #30390; available at https://www.caymanchem.com/product/30390). This meticulously assembled collection spans a wide array of compounds known for their antiviral properties, targeting various stages of viral life cycles such as entry, replication, and maturation. The library features compounds that are effective against a diverse group of viruses, including but not limited to HIV, hepatitis B (HBV) and C (HCV) viruses, influenza, herpes simplex virus (HSV), and various coronaviruses. In addition, it contains several agents with known antimalarial capabilities. The molecular targets of these compounds are varied, encompassing HIV reverse transcriptase, protease, integrase, RNA-dependent RNA polymerases, and the nonstructural proteins of HCV, among others. Supplied as 10 mM stock solutions in dimethyl sulfoxide (DMSO), the compounds were organized in 96-well plate format within Matrix tube racks. As delineated in the subsequent methods section, screening for toxicity and cytotoxicity was performed using the AsedaSciences SYSTEMETRIC® Cell Health Screen alongside developmental zebrafish assays.

### AsedaSciences SYSTEMTRIC® Cell Health Screen

In this study, the evaluation of antiviral compound cytotoxicity was conducted using phenotypic readouts from HL60 cells, a human promyelocytic leukemia cell line. The cells were cultured in suspension within 850 cm^2^ roller bottles that featured vented caps. The culture medium was RPMI 1640 without glucose, enriched with 10 mM galactose and 10% dialyzed, heat-inactivated fetal bovine serum (FBS). The cultures were maintained at a rotation speed of 1 RPM, in an atmosphere of 5% CO_2_ at a temperature of 37 °C.

For the cytotoxicity assays, the HL60 cells were distributed into a 384-well plate format. Each compound from the library was subjected to a ten-step, threefold serial dilution, covering a concentration range from 5 nM to 100 µM. The cells were exposed to these diluted compounds for 4 h at a temperature of 37 °C, under a 5% CO_2_ atmosphere. To ensure equilibrium between the serum components and the chemicals under study, the assay plates were pre-sealed and stored in a dark environment at room temperature for 2 h before the cell exposure.

Following exposure, the assay plates were briefly centrifuged at 300 g for 2 min to gently pellet the cells. Using a Biomek NXP automation workstation, 20 µL of the supernatant from each well was carefully aspirated, and subsequently, 20 µL of a fluorescent dye mixture was added to each well. The plates were then sealed and vigorously shaken twice at 2,200 RPM for 5 s each to ensure thorough mixing. The entire process of chemical treatment, cell dispensing, and dye addition was automated to ensure consistency and reproducibility. The final status for the assay was 100,000 cells per 40 µL volume in each well.

Data were collected using a CyAn™ ADP flow cytometer (Beckman Coulter), equipped with a HyperCyt® autosampler (Intellicyt) for automated sampling. Flow-cytometry data from each well, along with a detailed map of well contents, were uploaded to a cloud-based platform. An automated data-processing pipeline then performed quality control on each plate, followed by feature extraction and machine learning-based classification. This rapid and reproducible approach enabled efficient and accurate analysis of the cytotoxic and acute cellular stress effects of antiviral compounds on the HL60 cell line, offering valuable insights into their safety profiles.

### ZBEscreen™

#### Animals and exposures

Wildtype zebrafish (Tropical 5D) were housed at Oregon State University Sinnhuber Aquatic Research Laboratory (Corvallis, OR) as 1,000 fish per 100-gallon tank according to the Institutional Animal Care and Use Committee protocol (ACUP 2021–0227). The fish were maintained at 28 °C on a 14:10 light/dark cycle in recirculating water supplemented with Instant Ocean. Adult and juvenile fish were fed twice daily with appropriately sized Sparos food, while larvae were fed 3 times a day. Spawning funnels were placed in the tanks the night prior, and the following morning, embryos were collected and age-staged (Kimmel et al. 1995; Westerfield 2000). Embryos were maintained in embryo medium (EM) in an incubator at 28 °C until further processing. EM (pH 7.3) consisted of 15 mM NaCl, 0.5 mM KCl, 1 mM MgSO_4_, 0.15 mM KH_2_PO_4_, 0.05 mM Na_2_HPO_4_, and 0.7 mM NaHCO_3_ (Westerfield 2000).

An initial screen was conducted with 32 embryos per compound at an assumed maximum tolerable concentration of 100 µM to optimize throughput. Compounds that elicited significant morphologic changes were then subjected to comprehensive testing across a gradient of 11 concentrations (0, 0.005, 0.015, 0.046, 0.15, 0.41, 1.23, 3.7, 11.1, 33.3, 100 µM), to ascertain dose–response relationships. Parathion, a known teratogen in embryonic zebrafish, was included on each test plate as the positive control across a narrow concentration band (14.3, 19.3, 24.2, 29.1 µM), ensuring the reliability and sensitivity of the assay.

Embryos were dechorionated at 4 h post-fertilization (hpf) and singulated into 96 well plates as described in (Mandrell et al. 2012; Truong et al. 2014). All compounds and the parathion positive control were delivered to the plate wells preloaded with an embryo in 100 µL of EM using an HP D300 digital dispenser. The D300 dispensed all target concentrations directly from the 10 mM library stocks dissolved in dimethyl sulfoxide (DMSO), and direct from 10 -mM parathion in DMSO. Each antiviral test concentration was replicated in 7 wells per plate. Each parathion concentration was replicated in 3 wells per plate, and each plate contained 14 replicate wells of solvent control (embryo in EM + 1% DMSO). All plate wells were normalized to 1% DMSO by the D300 dispenser and each plate was replicated twice.

The plates were sealed against en masse fluid loss by a parafilm sheet between the plate and lid and incubated in darkness at 28° ± 1 °C. To ensure uniform chemical mixing, plates were placed on an orbital shaker at 235 RPM overnight until 24 hpf (Truong et al. 2016). At this stage, assessments were made for embryonic photomotor response (EPR), mortality, and abnormal morphology. The plates were returned to the incubator until 120 hpf when larval photomotor response (LPR), mortality, and morphology were assessed.

#### Developmental data curation

Mortality, morphology, EPR and LPR data were collected with a custom Laboratory Information Management System (LIMS), the Zebrafish Acquisition and Analysis Program (ZAAP), which utilizes a MySQL database. The data endpoints were associated via plate barcode with chemical and concentration metadata. Data analysis was via custom R scripts that processed data within the LIMS framework (Truong et al. 2014).

#### Mortality and morphology assessments

Mortality and developmental progress were assessed at 24 hpf. By 120 hpf, we counted new mortality and scored nine morphologic endpoints for abnormality as binary (normal/abnormal) outcomes: Cranial, axis, edema, muscle, lower trunk, brain, skin, notochord (Truong et al. 2022).

#### Embryonic photomotor response (EPR)

The EPR assay took place at 24 hpf (Reif et al. 2016; Truong et al. 2022). The test plates remained in darkness until the assay to avoid desensitizing the embryos to white light. The EPR was video captured under infrared light with photo-stimulation by two 1-s bursts of white light. These bursts were administered 30 and 40 s into the video recording, with the three periods immediately before and after these pulses considered the background, excitatory, and refractory periods. Photomotor activity during these intervals offered insights into the potential impacts of the chemicals, independent of visual input as the eyes are not functional at 24 hpf.

#### Larval photomotor response (LPR)

At 120 hpf, we assessed the LPR using Viewpoint Zebrabox movement tracking chambers and software. The assay was 24 min of 4 cycles of 3-min light: 3-min dark. The distance moved by each larva was integrated over 6 s time bins. Whereas EPR measured the hindbrain photoreceptor-triggered motor response, LPR measured a visual-triggered motor response.

### Statistical analysis

All analyzed data are provided in Table S1.

#### Morphology—maximum tolerable concentration

Statistical evaluation of the morphologic endpoints was carried out with custom scripts executed in R language for statistical computing (Team 2021). Following Truong et al. (2014), binary outcomes for each morphologic endpoint were extracted from the Zebrafish Acquisition and Analysis Program (ZAAP). A significance threshold was determined for each chemical–morphologic endpoint pair by comparing it to the incidence rate in the control group. The outcomes for each endpoint were binary (0 for absence, 1 for presence of an effect) and documented for every well, representing a sequence (n = 14) of Bernoulli trials for analysis.

#### Morphology—dose response

The presence of any morphologic changes was noted as a binary (0 for no effect, 1 for effect observed) response for each of 13 predefined endpoints. For analysis, these individual responses were aggregated into a single binary outcome termed ‘ANY’. To evaluate the morphological activity of the compounds, a Benchmark Dose (BMD) methodology was utilized, focusing on the ‘ANY’ endpoint through a parametric curve-fitting technique (Truong et al. 2022). Specifically, the data were analyzed using an unrestricted (parameters were not constrained) 3-parameter log-logistic model tailored for binary outcomes, incorporating an “extra risk” factor, in line with the Environmental Protection Agency’s Benchmark Dose Software (BMDS) version 3.2 guidelines (EPA 2020). The benchmark response (BMR) was established at a 10% deviation from the baseline response rate, and from this, the BMD_10_ values were determined.

#### Behavioral analysis—LELS

For the EPR assay, statistical significance was calculated for each interval: background, excitatory, and refractory using a Kolmogorov–Smirnov test (K–S with a threshold of *p* < 0.01Reif et al. 2016; Truong et al. 2022). The lowest concentration identified as statistically significant was set as the LEL for each interval. The LEL for each compound was determined by taking the lowest LEL across the intervals.

For LPR, only the last 3 cycles of light and dark were used for analysis. Any dead or malformed animals were removed from behavior analysis, and concentrations in which more than 30% of the individuals were malformed were also removed. Statistical significance was determined using a K–S test (*p* < 0.01) by combining all activity in the three light phases and repeating this for the three dark phases. The LEL for each phase was set to the lowest concentration with statistical significance. The LEL at the compound level for LPR was the lowest of the LEL for the phases.

#### ZBEscreen LELs

The morphology assay endpoint ‘ANY’, was reported as the BMC_10_ while the two behavioral endpoints were reported as an LEL. For comparison to the single CHS value, a ZBEscreen LEL was computed by taking the lowest value across all three assay endpoints.

### Self-organizing map

The SMILES file for each of the 403 compounds was merged with the ZBEscreen and CHI data as input into DataWarrior (openmolecules.org) (Sander et al. 2015). Using the six parameters (CHI, ZF.Hit, EPR, LPR.dark, LPR.Light and SMILES) as input, the response data (all but SMILES) was used in a Gaussian neighborhood function to create a 200 neurons per axis self-organizing map (SOM).

## Results and discussion

### Landscape of the 403 Cayman antiviral library

**Fig. 1:**
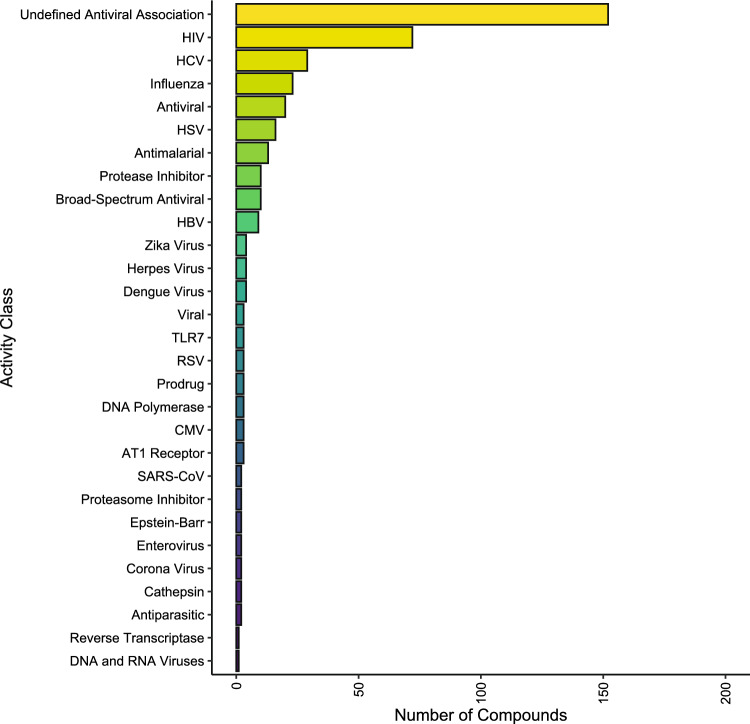
Landscape of Cayman antiviral library (403). Cayman documented the compounds mechanism of action into 29 activity classes, with the largest number of compounds belonging to the top 3: Undefined antiviral association, HIV, and HCV (151, 72, and 29, respectively)

The Cayman antiviral library contained 403 compounds categorized into 27 distinct groups based on their antiviral activity (Cayman Chemical Company 2023). The largest category was “Undefined antiviral association,” with 151 compounds or 37.5% of the library. The next largest group targeted human immunodeficiency virus (HIV) with 72 compounds or 17.9% of the library (Fig. 1). Other significant categories targeted Hepatitis B (HBV), Hepatitis C (HCV), influenza, HSV, or were antimalarials, broad-spectrum antivirals, protease inhibitors. Each ranged from 9 to 29 compounds, totaling 139 compounds or 34.5% of the library. The remaining 17 categories contained fewer than 4 compounds each.

The mechanisms of action for these compounds were divided into 17 classes, with inhibitors, undefined mechanisms, and prodrug inhibitors being the most prevalent, numbering 189, 137, and 28, respectively. Of the 403 compounds, 114 have undergone FDA evaluation, receiving designations such as prescription, discontinued, over the counter, or a combination of prescription and discontinued. Notably, 15% (17 compounds) have been discontinued for various conditions including hypercholesterolemia, hepatitis, Paget’s disease, and viral infections like HSV-1, HCV, HBV, and HIV. Three compounds were available over the counter, including Docosanol, Loratadine, and N-acetylcysteine. The 94 compounds currently prescribed targeted viral infections such as HIV, HCV, HSV, and HBV.

These compounds in the library are believed to operate through 17 distinct mechanisms, including activation, agonism, antagonism, antioxidation, chelation, DNA synthesis induction, and various types of inhibition. Among these were 189 inhibitors, 30 prodrugs, and 137 compounds with undefined mechanisms. Of the 137 with undefined mechanisms, 11 have been FDA-approved for malaria, osteoporosis, HBV infections, seizures, actinic keratosis, sickle cell anemia, intermittent claudication, and lupus, reflecting the library’s potential against a wide spectrum of diseases. HIV and HCV were the most targeted, by 27 and 12 compounds, respectively. The library especially emphasized HIV antiviral therapy, with 21 compounds currently prescribed and 6 discontinued.

A review of the Cayman antiviral library annotations indicated that 114 of 403 compounds (28.3%) were Food & Drug Administration (FDA) approved. Of these 17 compounds (4.2%) were previously approved by the FDA but are now discontinued (6 discontinued were treating HIV infections, 3 HCV), while only 2 compounds (0.5%) are currently available over the counter (OTC). Most (95 compounds, 23.6%) were prescription only. Most prescription compounds treated HIV and HCV (29), malaria (6), HBV (6) and influenza (3).

### 25% of Cayman library identified as toxic by CHS

Toxicity screening of a 403-compound antiviral library was performed using the Aseda Sciences Systematic Cell Health Screen (CHS), an in vitro assay designed to evaluate cellular stress across multiple parameters (Bieberich et al. 2021). The CHS assay quantitatively measures eight key cellular stress indicators: cell morphology, membrane integrity, reactive oxygen species (ROS) production, glutathione levels, nuclear membrane integrity (assessed via two distinct metrics), cell cycle progression, and mitochondrial membrane potential. These measurements are combined to generate a CHI, which estimates the toxicity potential of each compound, with higher CHI values (approaching one) suggesting increased toxicity. The CHI serves as a pseudo-probability metric that assesses how closely a compound’s phenotypic pattern matches those found in a training library of known toxic compounds with various mechanisms of action.

**Fig. 2:**
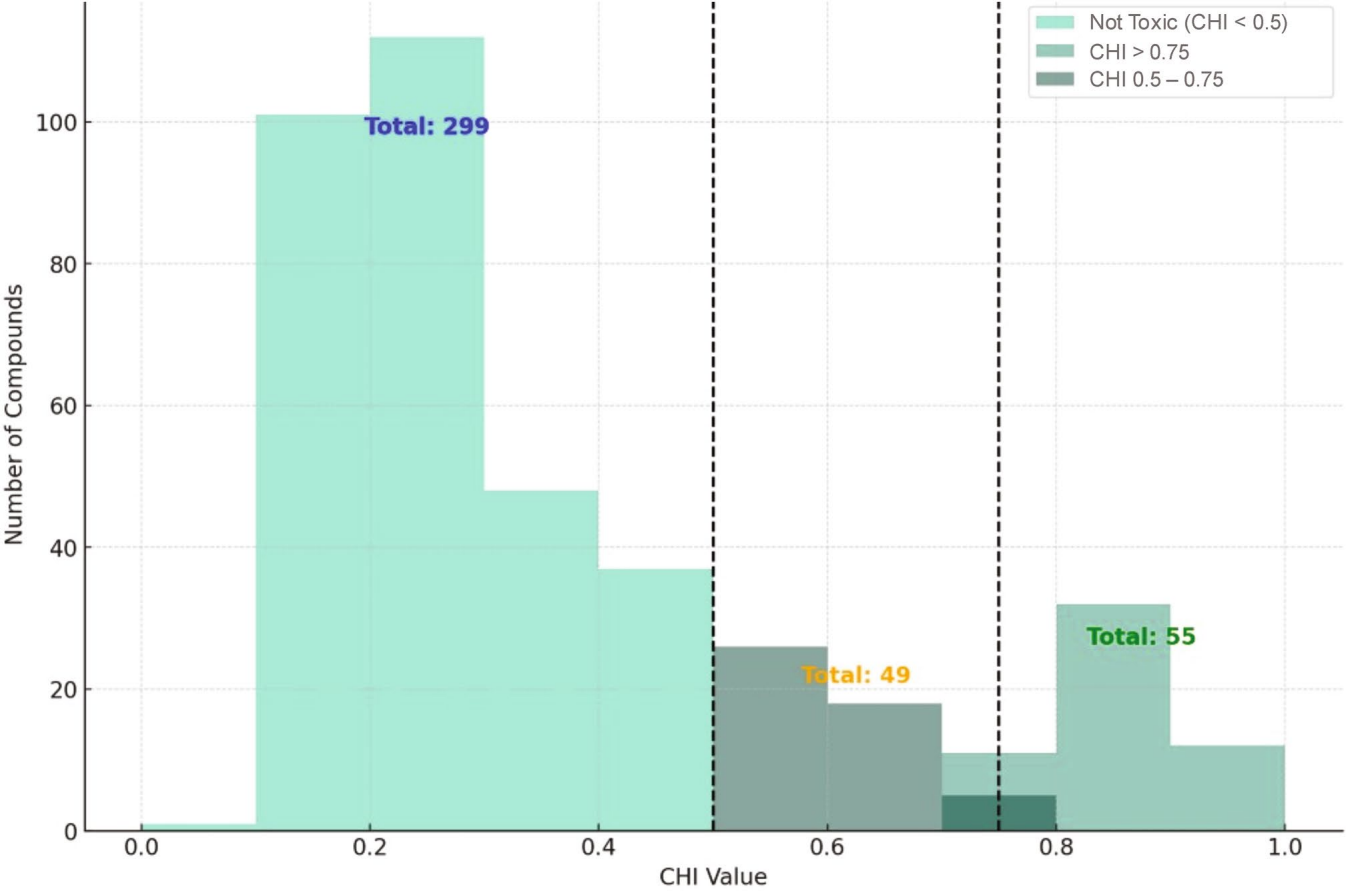
CHI breakdown for Cell Health Screen (CHS). The CHI is an indicator of toxicity for compounds. As CHI approaches 1, this indicates high toxicity. A CHI of > 0.5 has a higher probability of toxicity (104), and the most active are those with > 0.75 (55) to the right of the dotted line

The initial screening, conducted at ten different concentrations, indicated that 74.2% (299) of the compounds were low-risk, based on a CHI threshold of 0.5 (Fig. 2). Further analysis identified 104 compounds (approximately 25% of the library) as high-risk under this criterion. Applying a more stringent CHI threshold of > 0.75 narrowed the selection to 55 compounds. Saikosaponin A exhibited the highest toxicity potential (CHI = 0.958), followed closely by NH125 and gallotannin (CHI values of 0.95 and 0.935, respectively), all of which are plant-derived compounds used in the treatment of inflammatory conditions and cancer.

Further categorization of the activity of these 104 high-risk compounds was distributed across 27 distinct activity bins, with their mechanisms of action falling into six primary targets: agonists, antagonists, antioxidants, inhibitors, ligands, and prodrug inhibitors, with some remaining undefined. Most of these compounds were characterized as inhibitors (65) or had undefined targets (31), underscoring the prevalence of inhibition as a key mechanism of antiviral activity. Of the 189 total inhibitors in the library, the CHS assay identified 34% as toxic. Inhibition as an antiviral mechanism was not measured in this study. Still, an indirect association between inhibitor efficacy and developmental toxicity to zebrafish was plausible and may have been the driver of toxicity in this group.

### Developmental zebrafish identifies hazard associated with an additional 20% of Cayman library

**Fig. 3:**
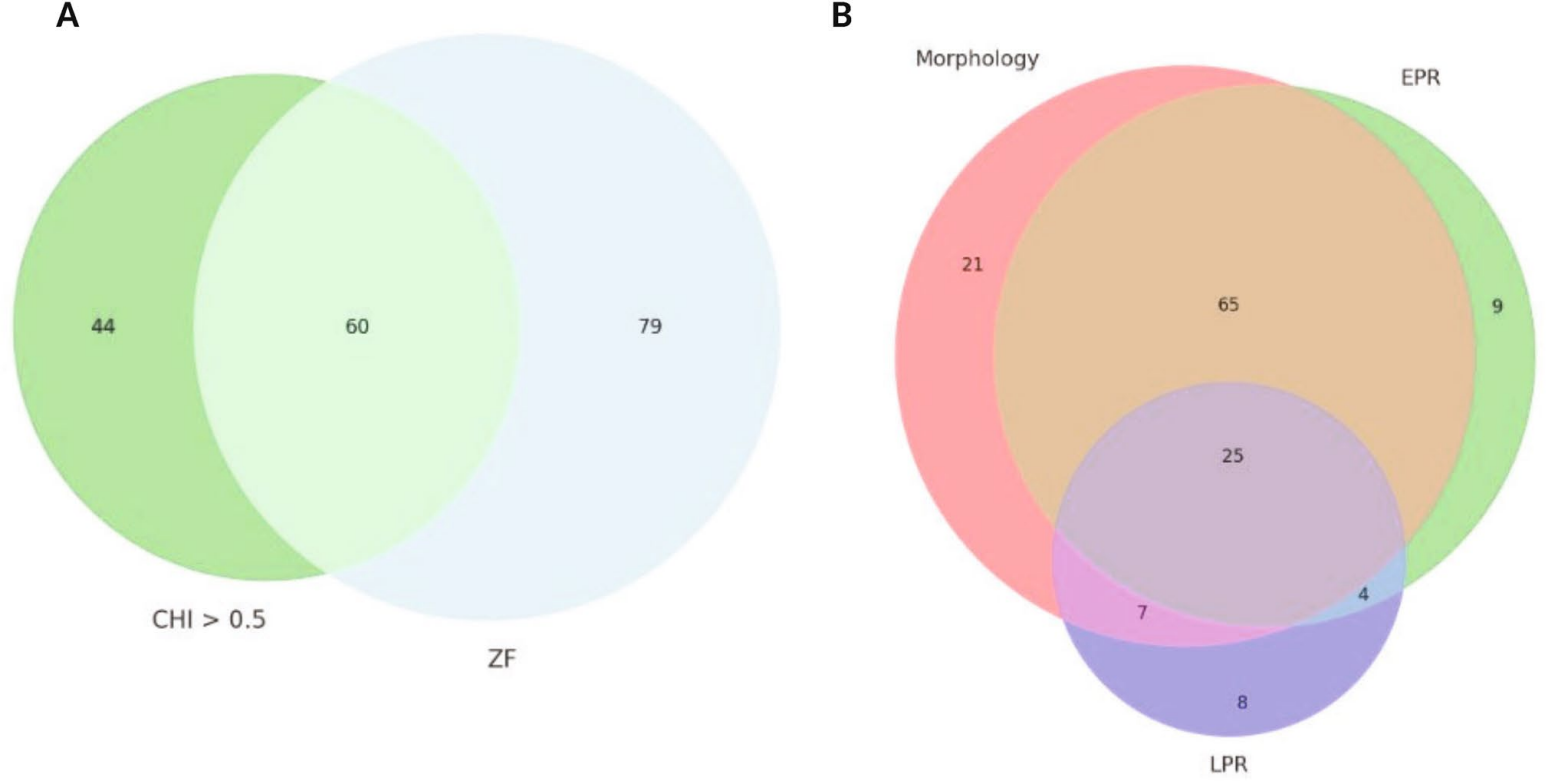
Bioactivity profiles. Comparison of the active calls in (**A**) between CHI > 0.5 and in zebrafish, and (**b**) breakdown of the zebrafish hits across the three assays

Of the 104 compounds that exhibited a CHI exceeding 0.5, ZBEscreen identified 60 (57.7%) as toxic, manifesting either adverse morphologic effects (DEVTOX) or aberrant embryonic and larval photomotor responses (EPR and LPR, respectively) (Fig. 3a; Table S2). The other 44 compounds were only a hit in CHS, but not in zebrafish. Of these 44, 10 were FDA and European Medical Association (EMA) approved — nine currently authorized for use by both, and one withdrawn by EMA (Doxycycline hyclate). These drugs were approved primarily for infections (HIV, HBV, HCV and bacterial) except for Dabigatran etexilate used for Venous thromboembolism. For five of these prescribed compounds, severe liver injury has been reported when taken in combination with other drugs, demonstrating that CHS correlates with in vivo toxicity. The lack of hit concordance between CHS screen and ZBEscreen can be attributed to differences in metabolism and compound solubility.

If the CHI cutoff was increased to 0.75, the zebrafish assay identified 34 compounds as toxic. It was observed that 49 compounds had CHI values ranging between 0.5 and 0.75, while 55 compounds exceeded a CHI of 0.75 (Fig. 2). The zebrafish developmental assay detected toxicity from an additional 10% of compounds (79 in total) that did not register as toxic in the CHS assay. Specifically, 64 out of the 79 compounds induced developmental toxicity, primarily evidenced by increased mortality rates. This demonstrates the advantages of the additional behavioral and developmental measurements that can be observed in a live, whole organism model, adding depth to the toxicity coverage when used in combination.

In aggregate, the embryonic zebrafish model flagged 139 compounds as hazardous. The study’s aim was to use the ZBEscreen to rapidly identify hazardous compounds for further mechanistic investigation. Initially, all 403 compounds were screened only at 100 µM and the 162 hits in that screen were subjected to concentration–response testing for abnormal morphology and behavior readouts. This approach facilitated the identification of a benchmark concentration (BMC) for further testing. The concentration–response testing of 162 hits yielded 139 toxic hits in at least one of the three assays (Fig. 3b) and 25 compounds that were a hit in all three assays. There were 21 compounds that only elicited morphologic defects or morbidity, nine that only elicited abnormal EPR behavior, and eight compounds that only elicited abnormal LPR behavior.

This refined approach to early hazard detection underscored the utility of combining cellular and whole-organism models to provide actionable safety data well upstream of mammalian and clinical testing.

### Advantages and limitations of using BMC_10_ in aebrafish toxicity screening

The Benchmark Concentration 10 (BMC_10_) is a widely used metric for regulating compounds, as it incorporates the concentration–response relationship and accounts for variability within the data, providing a robust estimate of the concentration at which a specific adverse effect is observed in 10% of the population. However, the BMC_10_ method requires extensive, high-quality concentration–response data and involves parametric statistical modeling, which can limit its practicality for routine screening compared to simpler approaches such as the Lowest Effect Level (LEL). Due to these constraints, BMC_10_ was applied to the morphologic data but not to the behavioral data. If the LEL had been used for morphology, an additional five compounds would have been identified as toxic. Of these, CHS would have detected 3 (Saikosaponin A, Tyrphostin A9, and 20(S)-Ginsenoside Rh2), none of which have documented antiviral activity.

**Fig. 4:**
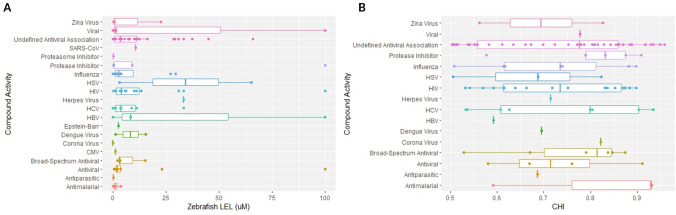
Distribution of compound activity across antiviral classes. Distribution of (**A**) Zebrafish Lethal Effective Concentration (LEL) and (**B**) Cytotoxicity Index (CHI) for different antiviral compound classes. The boxplot depicts the median (horizontal line within the box), interquartile range (IQR, represented by the box), and whiskers extending to 1.5 IQR from the median. Outliers are plotted as individual points

The ZBEscreen is multidimensional with several BMC/LELs for each compound, which made it difficult to compare to the CHS. To account for this, the ZBEscreen LEL was computed across all four of the assay endpoints. For the 139 zebrafish toxic compounds, 29 different antiviral compound classes were used (Fig. 4a). The class with the highest median ZBEscreen LEL was Herpes Virus with a median LEL of 75 µM. Thus, half of the compounds in this class had a ZBEscreen LEL higher than 75 µM, and the other half lower than 75 µM. The antiviral compound class with the lowest median ZBEscreen LEL was Broad-Spectrum Antivirals with a median LEL of 20 µM. These findings suggested that Herpes Virus compounds were less toxic than Broad-Spectrum Antiviral compounds.

For the 104 compounds with a CHI > 0.5, the antiviral compound class with the highest median CHI was Broad-Spectrum Antivirals (Fig. 4b). The median for this class was 0.85. The classes with the lowest median CHI were Dengue Virus and Corona Virus with median CHI approximately 0.7.

### Power of both model systems

In the Cayman antiviral library, 183 compounds (45.5% of the library) were hits in either or both assay systems. Most of these compounds had undefined antiviral associations (39.9%) and anti- HIV and -HCV made up the next two largest groups (17.5% and 7.7%, respectively) (Figure S1).

CHS detected toxicity from 44 compounds that ZBEscreen did not at 100 µM. The target activity of the 44 was primarily undefined (17) and secondary anti-HIV (13). The ZBEscreen detected toxicity associated with 79 compounds that CHS did not detect.

**Fig. 5:**
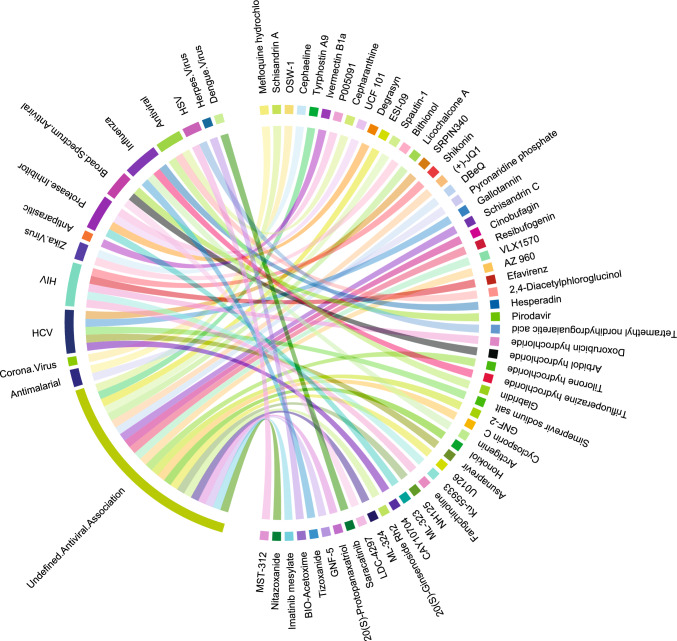
Inter-relationship between 60 common compounds hits between CHS and ZBEscreen and their activity class. A total of 60 compounds were toxic in both in vitro and in vivo and classified into 14 activity classes. Each color represents one of these parameters, and the line indicates the relationship between two parameters. The width of each parameter is a count of the number of relationships

Sixty compounds were active in both CHS and ZBEscreen (Fig. 5), with 23 compounds (38%) having an unknown mechanism. HIV and HCV (each with 5) were next, then influenza and protease inhibitors (4 each). Both broad-spectrum and general antivirals made up 6 members. The remainder, with 2 members or fewer, were anti-malarial, anti-zika, anti-HSV, anti-parasitic, anti-dengue, anti-coronavirus, anti-HBV, and anti-herpes.

The limited concordance between the CHS and ZBEscreen datasets was expected. The CHS assay is specifically designed to monitor rapid, acute responses for the parent compounds that serve as proxies for general acute cellular toxicity. In contrast, the ZBEscreen captures more complex and subtle interactions between toxic compounds and the physiology of whole organisms. While ZBEscreen is expected to detect many toxic effects identified by CHS, the reverse is less likely, as the cellular model used in CHS lacks the physiologic complexity and metabolic competency of the ZBEscreen to detect certain toxic actions. The physiologic dynamics of the processes occurring during vertebrate development cannot be replicated by even the most comprehensive set of in vitro assays. The CHS assay utilized HL60 cells, assessing 12 endpoints related to cell health, including cell morphology, membrane integrity, reactive oxygen species (ROS) production, glutathione depletion, nuclear membrane integrity, cell cycle progression, and mitochondrial membrane potential (Bieberich et al. 2021; St Mary et al. 2023)}. In contrast, ZBEscreen examined the outcomes of complex-signaling pathways over time, from early body patterning to metabolic competence and the interactions among tissues and organs during development. Although the developmental endpoints in ZBEscreen are less process-specific than the in vitro endpoints, they query a far broader range of biologic targets and events than CHS, while also incorporating metabolism and potentially capturing the toxic effect of metabolites. Metabolism is also a potential contributor to the differences between compounds toxic in the CHS screen versus negative in the ZBEscreen.

Moreover, the inclusion of photomotor behaviors in ZBEscreen introduces an additional dimension that cannot be replicated in vitro. The limited overlap between the two screens reflects their partially orthogonal designs and intended functions. Consequently, each system detected unique toxicities. When used together, these two screening approaches provide a more comprehensive method for detecting toxic effects than when either screen is used in isolation.

### Identifying key compounds with strong cellular impact through CHI and zebrafish assay analysis

There were 12 compounds with a CHI > 0.9, indicating an exceptionally detrimental impact on cell health. Notably, 11 of these 12 compounds were also hazardous in the zebrafish assay. The sole exception was Timosaponin AIII (CAS: 41059-79-4), a steroidal saponin that has potential therapeutic effects in cancer treatment, neuroprotection and anti-inflammatory roles. It has been used in traditional Chinese medicine but with a known cytotoxic potential (Han et al. 2018).

Four of the CHI > 0.9 compounds are established medications currently on the market: cepharanthine (CAS: 481-49-2), mefloquine hydrochloride (CAS: 51773-92-3), pyronaridine phosphate (CAS: 76748-86-2), and gallotannin (CAS: 5424-20-4) and all except mefloquine hydrochloride have demonstrated low toxicity profiles. Mefloquine hydrochloride, primarily used for malaria treatment, has shown potential for neurologic side effects in animal studies (Nevin 2014) including motor and respiratory impairment in small fish (Maaswinkel et al. 2015). Neurotoxicity was also observed in organotypic cultures (Yu et al. 2011).

Of the remaining seven highly toxic CHS compounds, six have yet to progress to market due to their toxicity in cell culture models. Cyclosporin C is structurally similar to Cyclosporin A, a known immunosuppressant on the market. The compound has promise for its potential to prevent organ transplant rejection and to treat autoimmune diseases (Ray et al. 2003). The last compound, simeprevir sodium salt (CAS: 1241946-89-3), was on the market to treat hepatitis, but was discontinued by FDA.

### Self-organizing map of the antiviral library

With the multi-dimensional data, a SOM with six parameters: CHI, zebrafish morphology, EPR, LPR-light, LPR-dark, and the SMILES for each compound, showed patterns and relationships from 200 neurons per axis.

**Fig. 6:**
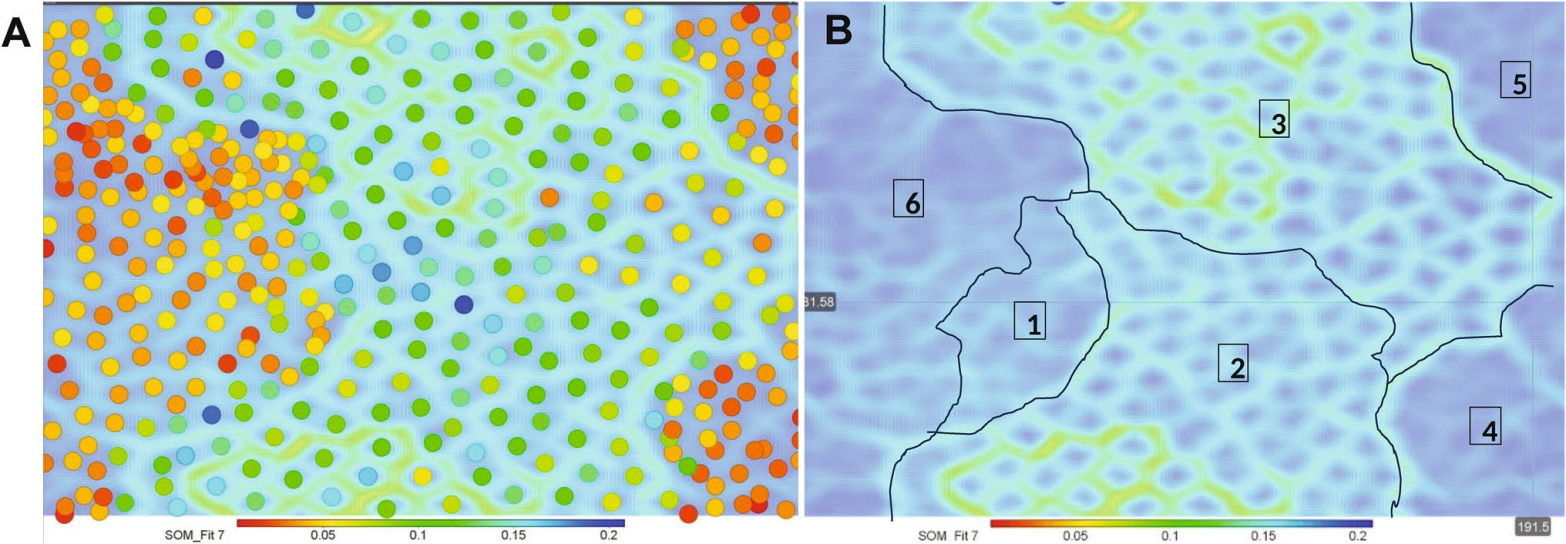
Self-organizing map. **A** SOM map displaying the fit quality for the ZBEscreen, CHI and the SMILES for each compound as nodes in the grid. Each hexagonal unit represents a SOM node, and the color intensity indicates the fit quality, where red indicates a poorer fit (higher error) and blue indicates a better fit (lower error). The hexagons are overlaid with circular markers representing individual samples, and the colors of the markers represent data point distribution over the map. Dense clusters of markers with a strong gradient between adjacent nodes. **B** The SOM with identified clusters outlined. The contours define specific regions of interest, indicating groupings of similar patterns across compounds. Neighborhoods 1 through 6 are marked, and the distinct boundaries between clusters demonstrate meaningful divisions. The color scale below the plot indicates the fit quality, with lower values (in blue) corresponding to better model fit

The SOM is an unsupervised data clustering methodology (Kohonen 1982). It has been widely used in drug screening and discovery applications (Tanrikulu et al. 2013). SOM_Fit values were computed and used as the color bar of Fig. 6A. They indicated a well-separated SOM (< 0.5) of 6 neighborhoods (Fig. 6B), which suggested that structure influenced both ZBEscreen and CHS outcomes. The largest neighborhood (blue and green background) was more dispersed, which suggested variability in the response and/or a range of compound structures that were less responsive in CHS and ZBEscreen. The neighborhood with mostly orange and red clusters represented a high density of similar compounds, which suggested conserved structure–activity relationships.

SOM neighborhood 1 illustrated the clustering of the most toxic compounds in the CHS assay. This neighborhood was highly connected with 2, showing those toxic in CHS and ZBEscreen. Neighborhood 3 compounds were toxic in zebrafish only, and those that were more densely clustered had more structural similarity. Neighborhoods 4, 5, and 6 were not toxic in the ZBEscreen but moderately toxic in CHS (CHI ~ 0.2), with neighborhood 6 having the most structural similarity.

Neighborhood 2 contained the most variable toxicity and the compound with the highest SOM_fit (0.5), arbidol hydrochloride (CAS: 1317070-25-0) with a CHI of 0.79 (morbidity, mortality and behavioral effects) in zebrafish at ~ 3 µM. Surrounding compounds had similar toxicity profiles such as shikonin (CHI 0.88) which elicited mortality in zebrafish at 3.46 µM by 24 hpf, and saikosaponin A (CHI 0.95) which elicited zebrafish mortality at ~ 3 µM by 24 hpf.

Arbidol hydrochloride is an antiviral drug known as umifenovir in Russia and China, for the treatment and prevention of influenza and oral viral infections (Boriskin et al. 2008). It inhibits viral fusion with the host cell membranes and targets hemagglutinin protein of influenza viruses, preventing the virus from entering host cells and inhibiting its replication. With the coronavirus pandemic in 2020, the COVID-19 antiviral efficacy of arbidol was evaluated in China (Zhao et al. 2022) where it was reported to accelerate the recovery of COVID-19 patients without significant adverse effects. In vitro test showed arbidol reduced hepatitis B virus DNA in extracellular supernatant (Li et al. 2021). It has not been approved by the US FDA or the European Medicines Agency (EMA), and the toxicity data reported herein suggest that the safety profile of arbidol may be considerably different from that of Chinese claims.

A neighbor to arbidol on the SOM was shikonin, a phytochemical with anti-inflammatory and antioxidant properties like arbidols. It is a major chemical component of Lithospermum erythrorhizon roots and may attenuate neuroinflammation and neurodegeneration (Liu et al. 2023). The compound has demonstrated neuroprotective properties in mouse brains attributed to its antioxidant properties (Wang et al. 2010). Shikonin can accumulate in mitochondria and generate ROS, resulting in a variety of adverse effects (Boulos et al. 2019), which may explain the toxicity observed in CHS and ZBEscreen. CHS measured mitochondrial membrane potential and impact to this endpoint would result in a higher CHI. Likewise, a potent inducer of mitochondrial ROS would be anticipated to adversely impact vertebrate development.

In the same SOM activity neighborhood, saikosaponin A was identified. It has traditionally been used in Chinese and Japanese medicine for modulating the immune system and as an anti-inflammatory (Lee et al. 2024) and is major component in the roots of Bupleurum sp. plants. Saikosaponin has antiviral, anti-fibrotic, and antioxidant properties and is used to treat liver disease, viral infections, and cancer therapy (Huang et al. 2024).

These three SOM neighbors were structurally dissimilar yet induced similar toxicity profiles and were anti-inflammatory and neuroprotective. This particular outcome did not support any sort of a predictive aspect of the dataset. However, assuming the growth of such a dataset to several thousand structurally diverse compounds, integrated structure–activity information would begin to enable hazard prediction.

### Zebrafish non-toxic library members were highly correlated with over-the-counter or prescribed antiviral drugs

We anticipated that OTCs and prescribed antivirals in the library would largely test benign in the ZBEscreen. Of the 263 compounds categorized as non-toxic, 241 were without morphological effects at 100 µM while 22 elicited abnormal behavior. The library had 114 compounds with FDA approval as “RX”, “OTC”, and 17 that were discontinued for various reasons after FDA approval (Figure S2A). Only three compounds were toxic hits in ZBEscreen: amodiaquine, simeprevir and probucol, each a discontinued drug. Amodiaquine was approved as a prodrug to treat malaria but was found to cause reproductive toxicity in rats (Niu et al. 2016). Simeprevir was approved to treat hepatitis C but was later found to cause severe liver toxicity in humans (Diseases 2012). Probucol was an approved antioxidant to treat hypercholesterolemia but was withdrawn in 1995 due to cardiotoxicity (Hirata 2021). Of the other 14 discontinued compounds, no adverse morphologic affects were observed at 100 µM and, because of this, they were not tested for abnormal behavior effects. Of the 17 discontinued drugs, only simeprevir was identified as a hit in CHS. The others had CHI values under 0.34.

From the library, 97 compounds, representing 24.1% of the total, are currently prescribed pharmaceuticals. Of these, 78 exhibited no morphological effects in the ZBEscreen while 19 were identified as toxic hits. A total of 17 were toxic in CHS with a CHI > 0.5. Seven of the currently prescribed pharmaceuticals were a toxic hit in both CHS and ZBEscreen. The compound with the highest CHI (0.93) was mefloquine, which has reported liver and CNS toxicity, although still used to treat malaria. In embryonic zebrafish, mefloquine had a BMC_10_ of 8.88 µM for ‘any effect’ and elicited abnormal swimming behavior at 3.7 µM. The other 6 currently prescribed but herein toxic hits in CHS also have known nervous system impacts (trifluoperazine, efavirenz, ivermectin), cardiotoxicity (doxorubicin, imatinib), gastrointestinal toxicity (niazoxanide). The only over-the-counter drug was loratadine, an antihistamine used to treat allergies. It was not identified as toxic in the CHS but was in zebrafish with a BMC_10_ of 1.23 µM. Other loratadine studies in developmental zebrafish demonstrated reduced heart rate and body length (Teixido et al. 2019) and altered cardiac β-adrenergic receptor activity (Steele et al. 2009). Decreased heart rate by competitive inhibition of muscarinic receptors in mammals has been noted when exposed to antihistaminic compounds.

Regulatory scrutiny by the EMA has been applied to 67 of these commercially available compounds, aligning with the FDA on 87% of the approval or withdrawal decisions (59 compounds). Within this subset, seven compounds were withdrawn from the European market. Only one compound discontinued by the FDA is still authorized for use by EMA (Figure S2B). Of eight chemicals discontinued by both agencies, only simeprevir was toxic in both CHS and ZBEscreen (CHI = 0.90, ZF any effect LEL = 3.60 uM). The other 7 had CHI < 0.34, and no LEL in zebrafish. During large randomized controlled trials of simeprevir, no link to increased instances of liver injury were detected, but after approval and wide scale use, a pattern of injury was observed. ZBEscreen and CHS both identified simeprevir as toxic. Perhaps it would never have made it to the clinical stage, let alone become a prescription drug, with better safety testing early in its development.

Of the 51 drugs approved by both EMA and FDA, 43 were non-toxic in ZBEscreen and 35 were non-toxic in CHS assays. The intersection of non-toxic compounds in both zebrafish and CHS assays underscored the thorough if inefficient, pre-market safety evaluation process. Those evaluations eventually got to the right place, but likely more slowly and at a much greater cost than the developers would have incurred had they availed themselves of the early testing we described here.

## Conclusion

Screening large compound libraries for drug lead discovery will probably continue well into the future. Our study demonstrated a way to rapidly rule out, or at least categorize as high-risk, compounds well upstream of the points where poor safety profiles have typically been discovered. A dual approach using the in vitro SYSTEMETRIC Cell Health Screen (CHS) and in vivo ZBEscreen together correctly distinguished known safe compounds from known hazards and those of unknown hazards among an antiviral library of 403 compounds. The developmental zebrafish test was more sensitive as it detected toxicity associated with an additional 20% of the library, activity that the CHS missed. The study identified 138 remaining compounds as having no detectable hazards. Our belief is that early application of this technique to any discovery library will greatly facilitate a better, faster, cheaper approach to lead discovery.

## Supplementary Information

Below is the link to the electronic supplementary material.

Supplementary file1 (DOCX 1038 KB)

Supplementary file2 (XLSX 97 KB)

Supplementary file3 (XLSX 24 KB)
