# Supplementary material for: Accelerating antiviral drug discovery: early hazard detection with a dual zebrafish and cell culture screen of a 403 compound library
Source: Arch Toxicol. 2024 Dec 27;99(3):1029–41. doi: 10.1007/s00204-024-03948-3 (PMC11821682; doi:10.1007/s00204-024-03948-3)
Supplement: Supplementary file 1 — Supplementary file1 (DOCX 1038 KB) [file 204_2024_3948_MOESM1_ESM.docx]

**Accelerating Antiviral Drug Discovery: Early Hazard Detection with a Dual Zebrafish and Cell Culture Screen of a 403 Compound Library**

Lisa Truong^1^, Andrew A. Bieberich^2^, Raymond O. Fatig III^2^, Bartek Rajwa^2,3^, Michael T. Simonich^1^, Robyn L. Tanguay^1^

1. Department of Environmental and Molecular Toxicology, Sinnhuber Aquatic Research laboratory, Oregon State University, Corvallis, OR 97333

2. AsedaSciences Inc., West Lafayette, Indiana

3. Bindley Bioscience Center, Purdue University, West Lafayette, IN 47907

**
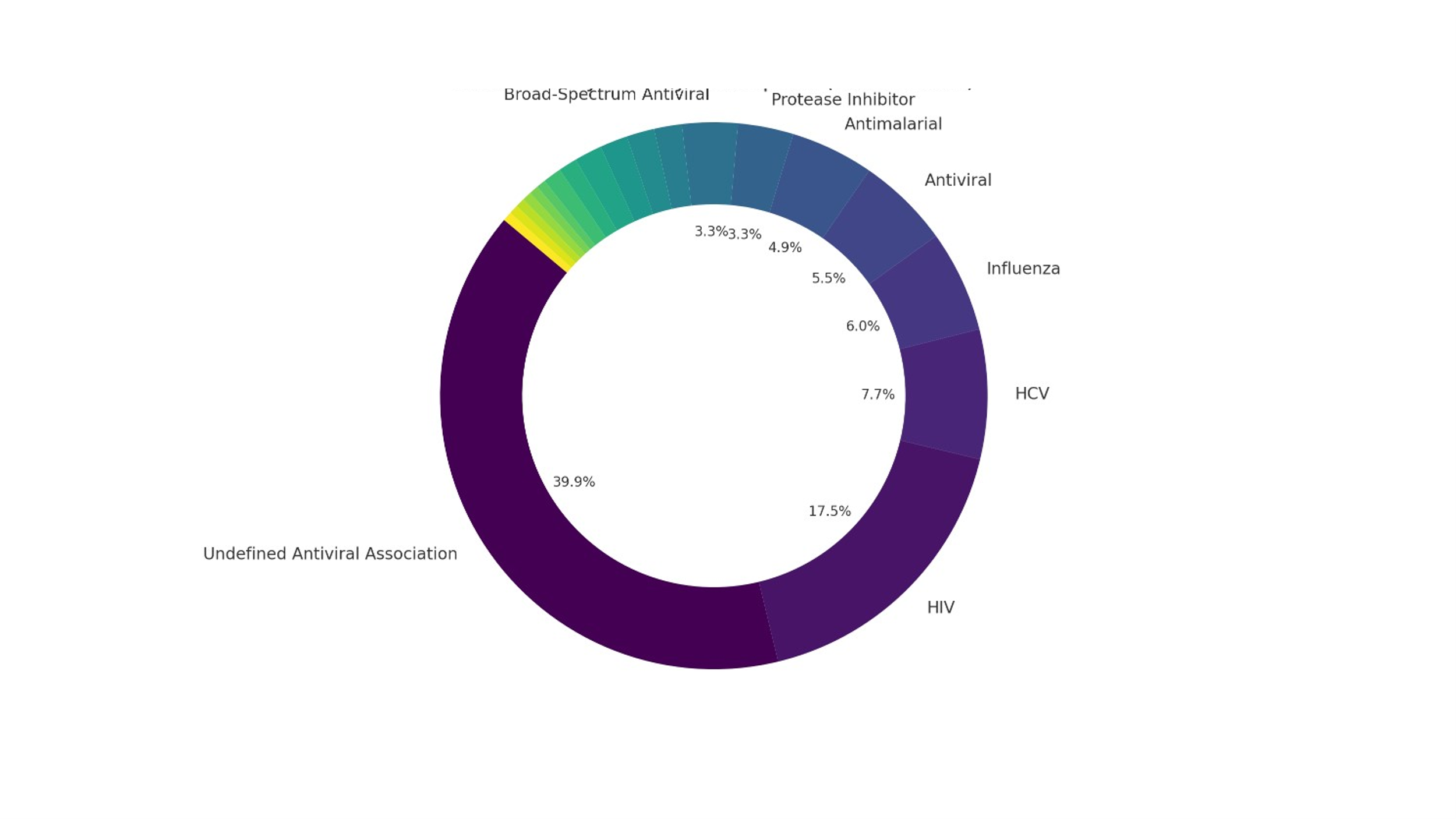
**

**Figure S1. Activity description of 183 antiviral compounds that were a hit in either or both model systems**

**
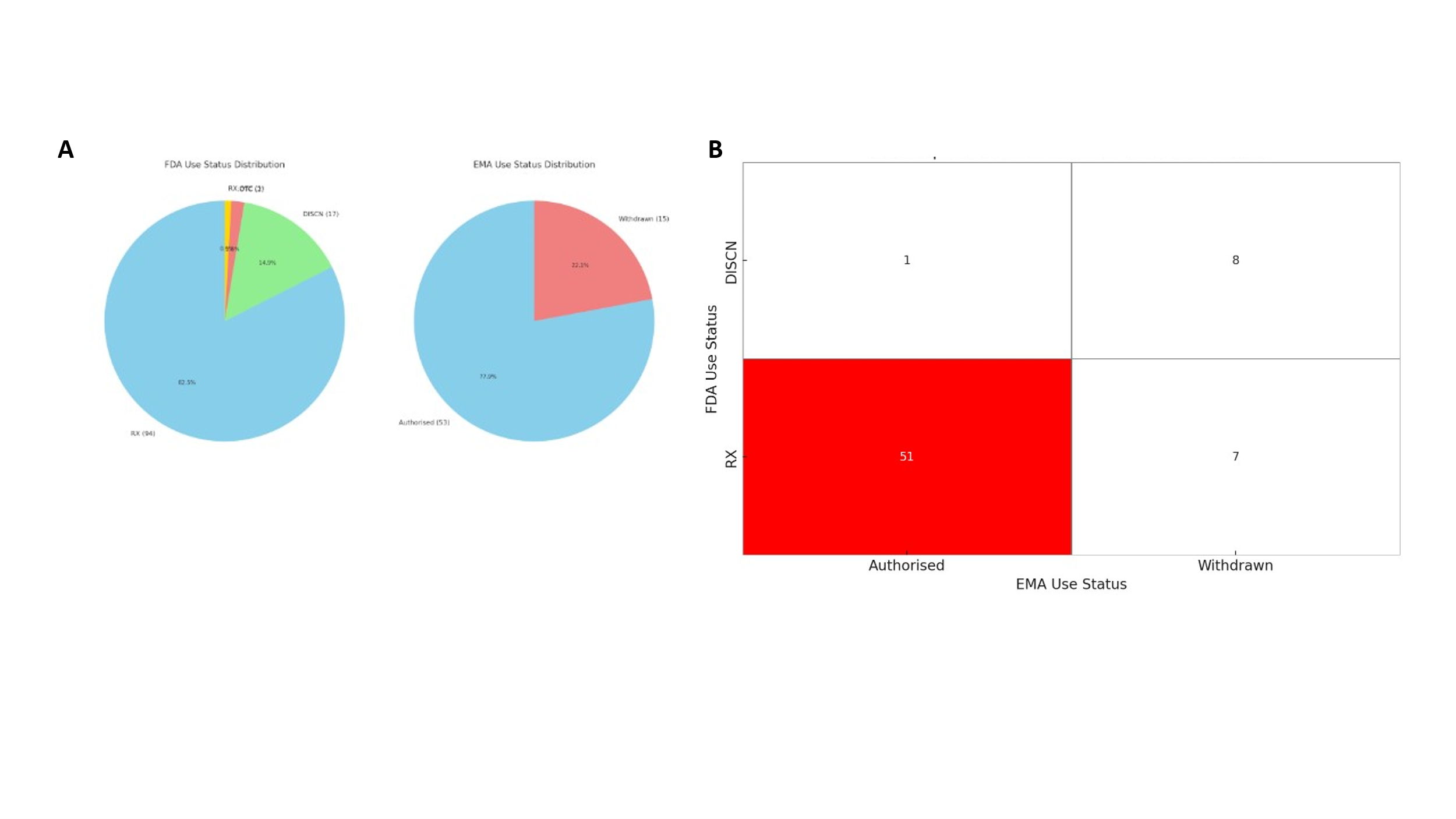
Figure S2. Distribution of the FDA/EMA approved drugs from the Cayman Library. (A)** 114 of the 403 compounds had FDA or EMA classifications. **(B)** Of the 114, there were 67 that were evaluated by both authorities, and only 51 (in red) were approved by both entities.
